# Study on the Effect of Temperature on Methane Catalytic Cracking over Biochar-Supported Fe

**DOI:** 10.3390/molecules31091479

**Published:** 2026-04-29

**Authors:** Xiye Chen, Jingdong Xu, Jiazhe Li, Lihua Zhu, Shipeng Sun, Xue Jiang, Feng Xu

**Affiliations:** School of Safety Engineering, Heilongjiang University of Science and Technology, Harbin 150022, China

**Keywords:** CH_4_, catalytic cracking, biochar-supported Fe catalyst, temperature

## Abstract

To achieve carbon neutrality, increasing efforts have been devoted to the clean utilization of fossil fuels. This study investigates the effect of reaction temperature on methane catalytic cracking over a biochar-supported iron catalyst. Corn stalks were heated to make biochar which was used as the carrier. To obtain biochar with a high specific surface area and well-developed porous structure, chemical activation was employed. The catalyst was made by adding iron to the biochar using the soaking method. This iron biochar catalyst is used to study its effectiveness in catalyzing methane cracking. The biochar-supported Fe catalyst was studied for its effectiveness in catalyzing methane cracking at different temperatures (800–950 °C). The results indicate that a higher temperature favors methane conversion in terms of reaction efficiency and cumulative conversion levels. At 950 °C, the catalyst exhibits the best performance, with a peak conversion rate of up to 85%, and it can still maintain a stable conversion rate of around 55% after prolonged reaction, yielding the total conversion of 57.6%. Raising the temperature can significantly promote the transformation of solid-phase products from highly blocking amorphous carbon to more ordered graphitized carbon. In addition, the reacted catalyst shows a remarkably reduced specific surface area, the disappearance of micropores, and a considerable increase in average pore size. Carbon nanotubes with various diameters and morphologies were formed on the catalyst surface.

## 1. Introduction

With the advancement of industrialization, global energy demand and reliance have been steadily increasing. In recent years, coal, oil, and natural gas have accounted for approximately 85% of total energy consumption. However, the overuse of fossil fuels has led to serious problems, including excessive carbon emissions and intensifying global warming [1]. These issues require urgent addressing and effective solutions.

Hydrogen is a clean and emerging energy carrier that could eventually reduce reliance on traditional fossil fuels. The byproduct of hydrogen is water vapor, which is environmentally benign. For these reasons, methods for producing hydrogen have attracted widespread attention from the scientific community [2]. Methods for producing hydrogen include coal gasification, biomass gasification, water electrolysis, and natural gas cracking [3,4]. According to statistical data, in the global hydrogen production mix, natural gas accounts for 48%, oil accounts for 30%, and coal accounts for 18% [5]. Natural gas remains essential in this field, as it is both cost-effective and supports the broader shift toward cleaner energy [6]. Methane makes up about 85% to 95% of natural gas. Currently, there are several routes for hydrogen production from methane. Hydrogen production methods like SMR, POM, and ATR emit large quantities of CO_2_, which conflicts with green development goals [7]. Methane cracking technology produces hydrogen and carbon materials, without generating carbon dioxide [8]. The carbon materials (such as carbon nanotubes and carbon fibers) exhibit excellent physicochemical properties and high added value, and can be widely applied in various fields [9]. Catalytic methane decomposition holds notable research significance and practical potential because it combines several key benefits [10]. It generates no CO_2_ emissions, features facile operability, enables convenient separation of hydrogen and solid carbon, and yields valuable solid products [11].

Catalytic methane decomposition is endothermic, requiring thermal input to drive the reaction. At lower temperatures, hydrogen production efficiency remains quite limited, while higher temperatures strongly promote methane conversion. Previous studies indicate that the equilibrium conversion of methane is only around 32% at 500 °C, but rises sharply to about 81% when the temperature reaches 700 °C [12]. However, under high-temperature conditions, catalyst deactivation occurs rapidly. Currently, catalyst deactivation in the methane catalytic decomposition process primarily stems from carbon deposition and sintering, which remains unresolved [13]. Methane decomposition catalysts are primarily classified by metal type into precious metal systems and non-precious metal systems, with the former including metals such as Pt, Ru, and Rh, and the latter encompassing Ni, Fe, Co, among others. Noble metals serve as highly active catalyst components with superior stability and catalytic performance [14]. Nevertheless, the high cost of noble metals and their severe sintering behavior at elevated reaction temperatures, which causes catalyst deactivation, restricts their application in large-scale industrial processes. In contrast, non-precious metal catalysts are generally more cost-effective and better suited for mass production and industrial utilization. Consequently, research interest in non-precious metal catalysts has been steadily increasing in recent years.

Fe-based catalysts have been extensively investigated among non-noble metal systems. Under high-temperature conditions ranging from 700 °C to 950 °C, this class of catalysts enables efficient catalytic decomposition of methane while maintaining stable reaction performance [15]. Jang et al. [16] prepared an Fe/Al_2_O_3_ catalyst to study the effect of temperature on the catalytic cracking of methane [12]. Their study revealed that the catalyst tends to deactivate rapidly at reaction temperatures below 750 °C. However, under higher reaction temperature conditions, the catalyst activity could be maintained for over 100 min. Their findings further indicated that during methane catalytic cracking at 1000 °C, the methane conversion increased progressively to 90% within 100 min of reaction.

One of the key research focuses in the field of catalysis is the design and development of biochar-based functional supports [17]. Biochar is a carbon-rich, porous material produced by the thermochemical conversion (pyrolysis) of renewable biomass feedstocks, such as agricultural residues, forest waste, or organic waste. Unlike fossil-derived carbon materials, biochar is a low-cost, sustainable product of waste valorization, with tunable physicochemical properties (e.g., surface area, pore structure, and surface functional groups) that can be tailored by adjusting pyrolysis temperature, atmosphere, and residence time. When biochar serves as a catalyst support, it can simultaneously provide catalytic functionality and fulfill the role of a support material [18]. The introduction of this support promotes the dispersion of Fe species and effectively inhibits high-temperature sintering of the catalyst [19]. On this basis, the incorporation of metals further enhances the efficiency of methane decomposition reactions [20,21]. Biomass is an abundant source and a clean and renewable energy source [22]. Benefiting from its well-developed porosity, low cost, and the wide availability of raw feedstocks, biochar has been extensively used as a catalyst support in heterogeneous catalysis. Investigating the functional role of biochar as a catalyst support holds considerable frontier significance [23]. Recently, biochar and metal-loaded biochar have also been explored as efficient catalysts or catalytic supports for hydrogen production and methane conversion [24]. The porous structure and surface defects of biochar favor the dispersion of metal nanoparticles and modulate the electron density of active sites, thereby improving catalytic activity and stability toward hydrogen production reactions [25].

Therefore, this study employs biochar-supported metallic Fe as the catalyst to investigate the effects of reaction temperature (800 °C, 850 °C, 900 °C, and 950 °C) on the characteristics of methane catalytic decomposition, through characterization of methane conversion and the physicochemical structure of the solid-phase products.

## 2. Results and Discussion

### 2.1. Effect of Pyrolysis Temperature on Reactivity

**Figure 1:**
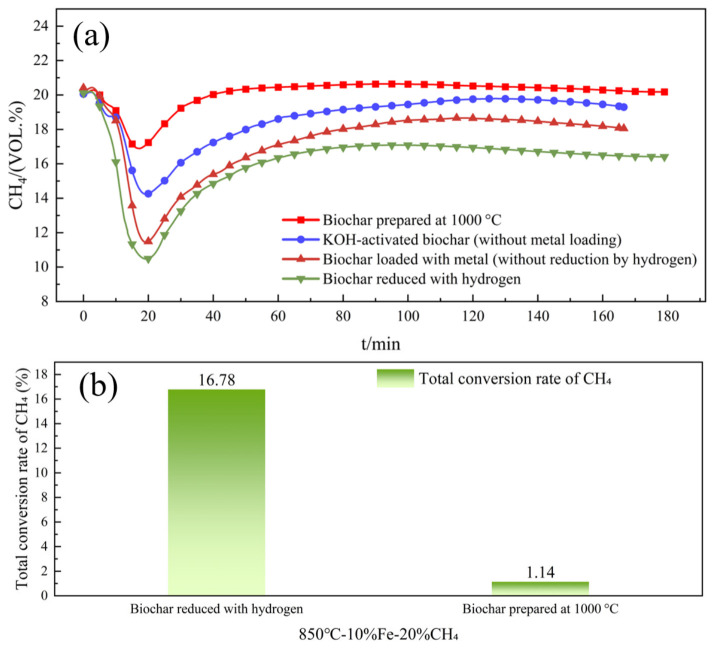
Catalytic performance of different biochar-based catalysts for methane decomposition: (**a**) CH_4_ concentration profiles with reaction time; (**b**) total CH_4_ conversion of pristine biochar and hydrogen-reduced metal-loaded biochar.

To elucidate the effects of KOH activation, metal loading, and hydrogen reduction on the catalytic performance, control experiments were conducted using pristine biochar prepared at 1000 °C and KOH-activated biochar (without metal loading). The results are presented in Figure 1a,b.

As shown in Figure 1a, pristine biochar exhibited negligible catalytic activity for methane decomposition, with only a slight decrease in CH_4_ concentration throughout the reaction. Correspondingly, the overall CH_4_ conversion rate of pristine biochar was only 1.14% (see Figure 1b), indicating that pristine biochar alone cannot effectively catalyze methane decomposition under the tested conditions. After KOH activation, the biochar showed a modest improvement due to its increased specific surface area and porous structure, yet its activity remained far lower than that of the metal-loaded catalysts. In contrast, the hydrogen-reduced Fe-loaded biochar catalyst demonstrated significantly enhanced catalytic performance: the CH_4_ concentration dropped sharply to approximately 10.2 vol.% within 20 min, achieving an overall conversion rate of 16.78%, which is more than 14 times higher than that of the pristine biochar.

These results clearly demonstrate that the introduction of Fe active sites and subsequent hydrogen reduction are crucial for activating the catalytic cracking activity. The pristine biochar and KOH-activated biochar alone lack the necessary active sites to promote methane decomposition, highlighting the critical role of metal loading in this system.

**Figure 2:**
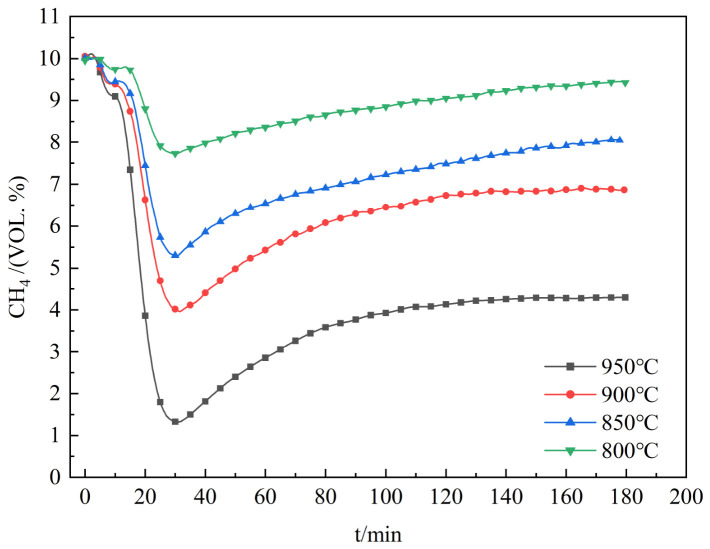
Variation Curves of Methane Volume Concentration at Different Temperatures.

Figure 2 illustrates the variation in methane volume fraction in the tail gas with reaction time at different temperatures. Real-time monitoring of the reaction products showed that in all cases, the methane concentration dropped rapidly at first, hitting its lowest point around 30 min. After that, it began to climb gradually and slowly stabilize. These results suggest that at the beginning of the reaction, the active sites of catalyst are fully available, which allowed methane molecules to be readily adsorbed and broken down, thereby rapidly reducing the methane concentration [26]. During the reaction, carbon from the methane covers the active sites [27], leading to the deactivation of the Fe-based catalyst, and consequently a gradual decline in reaction rate.

When the reaction temperature was set to 800 °C, 850 °C, 900 °C, and 950 °C, the lowest methane concentrations recorded were about 7.73%, 5.29%, 3.96%, and 1.32%, respectively. By the end of the 180 min reaction, the final methane concentrations at these temperatures reached 9.42%, 8.05%, 6.87%, and 4.30%. With increasing temperature, the activity of the catalyst active sites is significantly enhanced, which promotes the adsorption of CH_4_ molecules on the catalyst surface and accelerates the reaction rate. At 800 °C, the reactivity of methane is low. At 850 °C, its reactivity improves greatly. Therefore, efficient methane decomposition over the biochar-supported Fe catalyst requires a reaction temperature above 800 °C.

**Figure 3:**
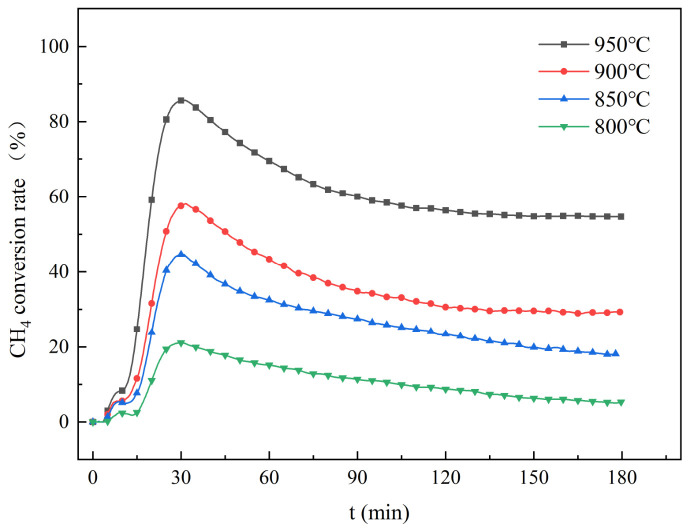
Methane conversion curves at different temperatures.

The methane conversion curves at different temperatures are shown in Figure 3. In all cases, the conversion increases rapidly at first, reaches a maximum, then declines gradually and eventually plateaus. This trend can be attributed to the gradual deactivation of the catalyst during the reaction. Under different reaction temperatures (800–950 °C), the methane conversion reached maximum values of roughly 21.1%, 44.7%, 58.1%, and 85.6%. After running for 180 min, the total methane conversion calculated over the full three-hour period came to 5.3%, 18.1%, 29.3%, and 54.7% under the same temperature conditions. Reaction temperature plays a dominant role in determining methane conversion efficiency [28]. High temperatures can greatly increase the conversion rate. They also help maintain stable conversion levels over time. The decrease in methane conversion later in the reaction is mainly due to the loss of the catalyst’s active sites. Possible reasons include the sintering of metal particles and carbon deposits covering the active surface. Although the catalyst undergoes gradual deactivation over time, it still exhibits stable performance and maintains a high methane conversion rate at 950 °C.

**Figure 4:**
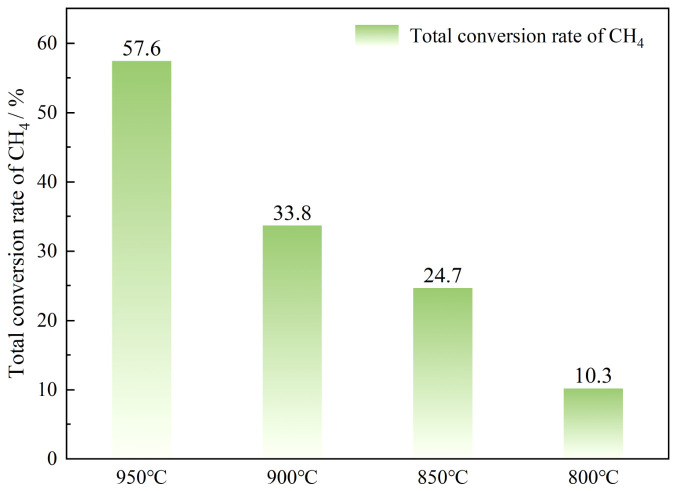
Overall methane conversion at different temperatures.

Figure 4 shows the total methane conversion after 180 min of reaction at different temperatures. Under an operating temperature of 800 °C, the total conversion of methane was only 10.3%, indicating that the ability to crack methane is limited at low temperatures. At 850 °C, the conversion rate improved to 24.7%. Although that is a significant improvement compared to 800 °C, it is still insufficient to meet the requirements for efficient conversion. As the temperature continued to rise to 900 °C and 950 °C, the methane conversion rates reached 33.8% and 57.6%, respectively. These results indicate that the methane decomposition reaction had already entered an efficient range at 950 °C, and the increase in temperature significantly enhanced the cracking efficiency of methane. In general, the higher the temperature, the better the overall conversion of methane. Especially at 950 °C, the catalyst delivered the best methane conversion performance, confirming that elevated temperature not only speeded up the reaction but also significantly enhanced cumulative conversion over time. Notably, the difference in total conversion between 950 °C and 900 °C was as high as 23.8%, far exceeding the increase of 9.1% between 900 °C and 850 °C, suggesting that once the temperature exceeded a certain threshold, the rate of the catalytic cracking reaction increased substantially.

### 2.2. Analysis of Carbon Materials

#### 2.2.1. Specific Surface Area and Porosity Analysis (BET)

**Figure 5:**
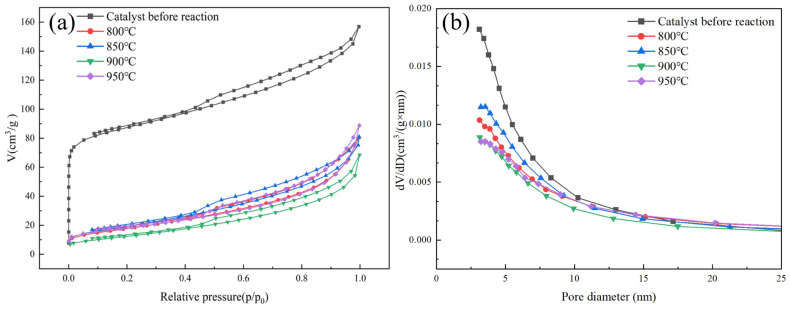
Analysis of specific surface area and porosity structure: (**a**) N_2_ adsorption–desorption isotherms, (**b**) pore size distribution.

Figure 5 shows the analytical results for specific surface area and pore characteristics. Before the reaction, the biochar-supported Fe catalyst exhibited a type IV adsorption–desorption isotherm accompanied by a distinct H_4_ hysteresis loop, indicating a well-developed micro-mesoporous composite pore structure. According to Table 1, the specific surface area reached as high as 327 m^2^/g, with a micropore area of 211.91 m^2^/g. These data indicate that a large number of microporous structures were formed in the biochar carrier during the preparation process, which provided a good foundation for the dispersion of iron particles and the adsorption of methane gas.

However, after the catalyst participated in the methane decomposition reaction, significant changes were observed in its specific surface area and pore volume. After the catalytic cracking reaction of methane was completed, characterization of the catalyst revealed a significant decrease in specific surface area. Micropores disappeared, mesopores decreased slightly, and the average pore size increased significantly. Carbon deposition generated during methane cracking was likely the primary cause of the reduction in specific surface area [29]. During the reaction, micropores were initially blocked or covered by carbon deposits, leading to a sharp decline in micropore volume and micropore area, thereby reducing the overall specific surface area. In addition, prolonged exposure to high temperatures may also cause the collapse of the pore structure within the biochar itself, further decreasing the specific surface area. The increase in pore diameter suggested that carbon deposited at high temperatures tends to form graphitized or fibrous carbon structures. The growth mechanism of such carbon promotes the expansion or reconstruction of pore walls, resulting in an increase in mesopore proportion and making it difficult to retain the original micropore structure. The study found that the reaction temperature altered the specific surface area and pore volume of the catalysts, yet the trend of its influence remained unclear [30].

**Figure 6:**
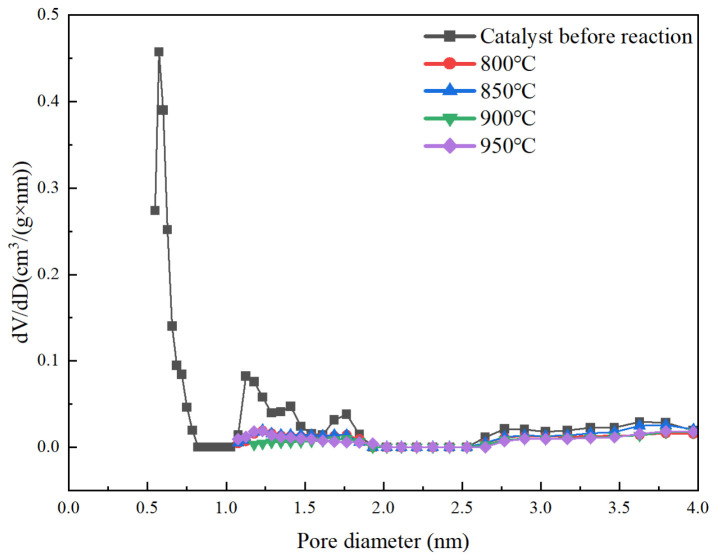
DFT micropore size distribution plot.

Figure 6 shows the micropore size distribution plot derived from DFT analysis. The DFT pore size distribution clearly shows that the catalyst before the reaction contains abundant micropores, which is consistent with the high micropore volume and surface area obtained from the t-plot method. After high-temperature reaction (≥800 °C), the micropore signal completely disappears, indicating the collapse of the microporous structure. The weak signals observed in the 1–2 nm range for the high-temperature samples (800–950 °C) are attributed to instrumental baseline noise and the lower detection limit of the DFT method, rather than real micropores. This is confirmed by the t-plot analysis, which shows zero micropore volume and area for these samples, indicating complete collapse of the microporous structure after high-temperature reactions. The negligible contribution of these signals to the total pore volume does not affect the conclusion of micropore disappearance.

#### 2.2.2. Scanning Electron Microscopy Analysis (SEM)

**Figure 7:**
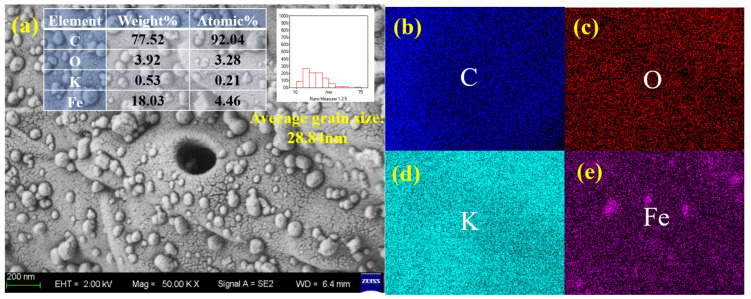
SEM-EDS scan image of biomass carbon loaded Fe metal catalyst before reaction. (**a**) The proportion of C, O, K, and Fe elements. (**b**–**e**) Distribution of C, O, K, and Fe elements.

The SEM-EDS characterization of biomass carbon loaded iron metal catalyst before methane cracking reaction is shown in Figure 7. Figure 7a shows that the catalyst surface exhibits irregular particle aggregation morphology. The quantitative and qualitative analysis data of EDS elements indicate that the dominant element in the catalyst system is carbon (C), which accounts for 77.52% of the total mass. The mass proportion of active metal Fe is 18.03 wt%. This is consistent with the theoretical impregnation loading of the catalyst (20% Fe). The EDS elemental mapping images in Figure 7b–e directly visualize the distribution of C, K, Ni, and O elements.

**Figure 8:**
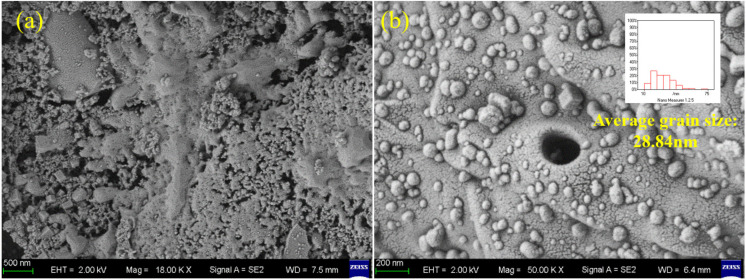
SEM characterization of the catalyst before reaction: (**a**) pore size distribution of the catalysts; (**b**) dispersion of iron particles in the catalyst.

SEM analysis was performed on the reduced catalyst sample, and the results are shown in Figure 8. From Figure 8a, it can be seen that the biochar support possessed a well-developed hierarchical pore structure. These pores created abundant specific surface area and void structure on the catalyst surface, which facilitated the adsorption and mass transfer of methane molecules as the reactant [31]. Fe nanoparticles were highly dispersed on the surface and inside the pores of the biochar. The average particle size of the active metal was determined to be 28.84 nm through analysis using Nano Measurer software (Version 1.2). Constrained by the mesopore size of the biochar, the iron particles generated during the reduction process at catalyst before reaction are relatively fine.

**Figure 9:**
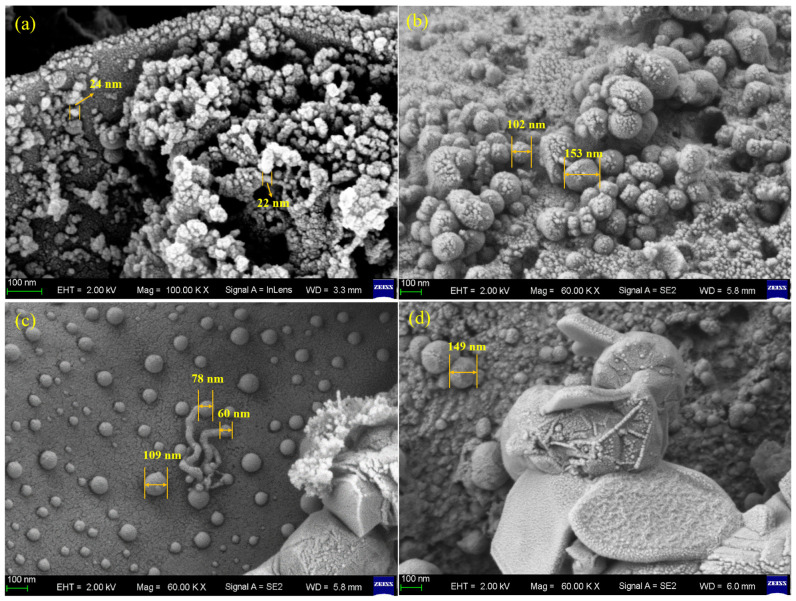
Morphology of the catalyst after reaction at different temperatures: (**a**) after reaction at 800 °C; (**b**) after reaction at 850 °C; (**c**) after reaction at 900 °C; (**d**) after reaction at 950 °C.

Figure 9a–d presented the topographical features of catalyst surfaces subsequent to methane cracking at multiple temperature conditions (800–950 °C). From the image, metal particles and carbon deposits can be seen. These structures changed when the temperature changed, which affected the performance of the catalyst. At 800 °C, the metal particles were highly dispersed, and the support retained abundant porous structures. The catalyst surface exhibited numerous uniformly distributed particles with relatively small sizes and minimal agglomeration, indicating limited pore blocking in the biochar. At 850 °C, the metal particles noticeably coarsened and began to aggregate locally. Spherical carbon deposits appeared on the surface, suggesting that carbon nucleated and grew epitaxially from the metal particle surfaces. The catalyst surface underwent pronounced changes at 900 °C, where large granular carbon particles formed, presenting densely packed spherical aggregates. Filamentous or tubular carbon structures can also be observed. At 950 °C, the catalyst surface was extensively covered by thick carbon deposits. In some regions, high-density carbon clusters and layered carbon were evident, which likely corresponded to graphitized carbon formed under high-temperature conditions.

#### 2.2.3. X-Ray Diffraction (XRD) Analysis

**Figure 10:**
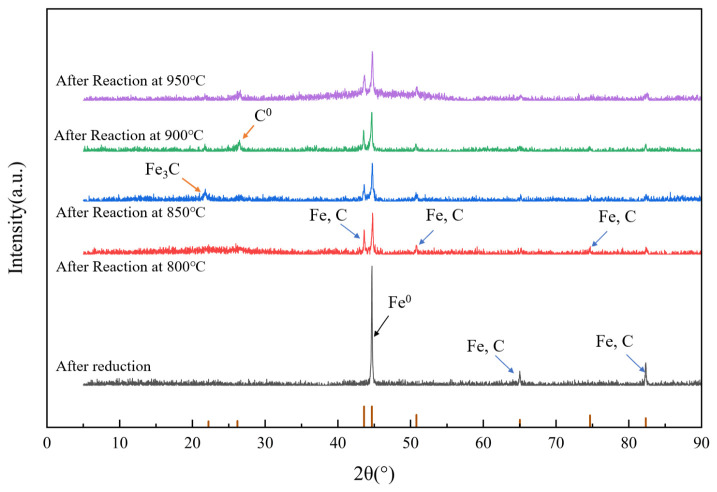
XRD patterns after reduction and reaction at different temperatures.

XRD patterns after reduction and after reaction at different reaction temperatures are shown in Figure 10. According to the XRD pattern of the reduced catalyst, a Fe^0^ peak was found at 44.6°, while peaks at 65.0° and 82.3° corresponded to austenite (a non-magnetic solid solution of carbon in γ-Fe). This indicated that iron in the biochar-based catalyst primarily existed in the form of zero-valent iron and austenite, with the formation of the latter potentially influenced by the loading amount and calcination conditions [31]. The XRD patterns of the post-reaction biochar-based catalyst showed dual diffraction peaks at 21.5° and 26.4°, corresponding to Fe_3_C and carbon, respectively. This demonstrated that after methane decomposition, the solid-phase carbon products primarily consisted of elemental carbon and Fe_3_C. Solid carbon predominantly adhered to the catalyst surface, continuously growing and depositing over extended reaction time. In addition, the weak diffraction peaks of Fe_3_O_4_ can be identified near 37.8°, 43.3° and 63.0°in the spectrum, indicating that the reduction process is incomplete, and some Fe still exists in the form of oxides. This mixed valence state of Fe^0^/Fe_3_O_4_/Fe_3_C is a typical feature of biochar-supported iron-based catalysts, which is conducive to redox cycle and electron transfer. When the catalyst participated in the methane cracking reaction, its overall phase structure changed significantly. The most significant change is reflected in the vicinity of 2θ = 26.4°, where a broad and obvious diffraction peak appears, which belongs to the characteristic signal of the typical (002) crystal plane of graphitized carbon. The appearance of this characteristic peak not only confirmed that a large amount of solid carbon produced by methane pyrolysis was successfully deposited in the solid phase of the catalyst, but that eventually part of the carbon interacted with Fe to form Fe_3_C. Carbon oversaturation may be a key factor in catalyst deactivation. The formation of Fe_3_C may also contribute to this deactivation.

#### 2.2.4. Raman Spectroscopy Analysis

**Figure 11:**
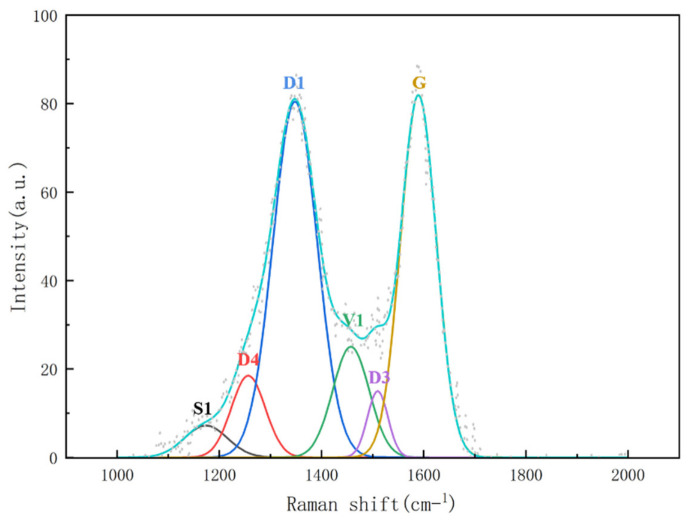
Peak deconvolution and fitting of the Raman spectrum (1000–2000 cm^−1^ region) for the catalyst sample.

In order to investigate the variation in structural ordering of carbon generated from methane decomposition at different reaction temperatures, Raman spectroscopy was performed on the post-reaction biochar-supported Fe-based catalyst samples over the wavenumber range of 1000–2000 cm^−1^. Subsequent peak deconvolution and fitting were employed to separate individual vibrational components, and the relative contributions of these components were studied based on the area ratios of the fitted peaks. The Raman spectra in the 1000–2000 cm^−1^ region were deconvoluted and fitted, revealing six distinct sub-peaks corresponding to different carbon structures. An example of the peak-fitting results for one sample is illustrated in Figure 11. The positions and assignments of the different sub-peaks are briefly described in Table 2 [32,33].

The G band originates from the in-plane C=C stretching vibration of ordered sp^2^-hybridized carbon hexagons and is commonly used as a characteristic peak for graphite or graphitic-like carbon. The presence of this peak indicates the formation of a certain degree of graphitized carbon (e.g., graphitic shells encapsulating metal particles). The D1-band represents a typical defect-induced peak, originating from the activation of aromatic ring breathing vibrations at lattice defects, edge sites, or in small crystallites. It is a key indicator for evaluating graphitization degree and defect density. Additionally, two broad and weak characteristic peaks of amorphous carbon were observed in the samples. The D3-band is generally assigned to C=C stretching vibrations in disordered sp^2^ carbon, associated with small aromatic clusters or partially graphitized but highly disordered carbon structures. The D_4_-band reflects vibrations related to heteroatoms such as C-C or C-O in mixed sp^2^/sp^3^ configurations, often attributed to the amorphous matrix derived from biomass carbon, cross-linked structures, and residual oxygen-containing functional groups. This suggests that the biochar carbon support retains a certain degree of disordered structural features. The *I_D_*_1_*/I_G_* ratio serves as a key indicator for characterizing the graphitization degree and defect density of carbon materials, with a lower value typically reflecting higher structural ordering and a greater degree of graphitization.

**Figure 12:**
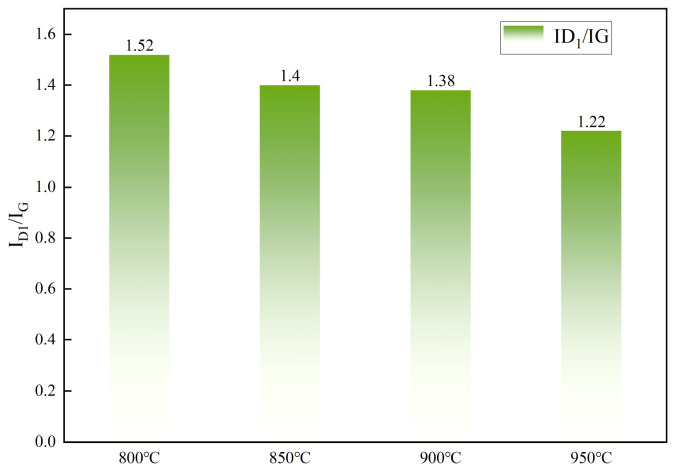
*I_D_*_1_*/I_G_* ratios obtained from Raman peak deconvolution and fitting at different temperatures.

The peak area ratios of the D-band to G-band of the catalysts after reaction at different temperatures are shown in Figure 12. With increasing reaction temperature, the *I_D_*_1_*/I_G_* value exhibited a clear monotonic decreasing trend, gradually declining from 1.52 at 800 °C to 1.22 at 950 °C. At 800 °C, the *I_D_*_1_*/I_G_* value measured was 1.52, which was relatively high. Such a high value suggested that methane cracking yielded predominantly amorphous carbon with a substantially disordered arrangement. As the operating temperature was increased to 850 °C and 900 °C, the *I_D_*_1_*/I_G_* values decreased to 1.40 and 1.38, respectively, suggesting an enhancement in the structural ordering of the deposited carbon species. At 950 °C, the *I_D_*_1_*/I_G_* ratio reached its lowest value of 1.22. This result was consistent with the data shown in Figure 8. Raman spectroscopy analysis showed that temperature was the key factor in determining the structure of carbon deposits from methane cracking. Increasing the temperature accelerated this structural transformation. The solid product transformed from disordered carbon into a more ordered, graphite-like form.

#### 2.2.5. Transmission Electron Microscopy Analysis (TEM)

**Figure 13:**
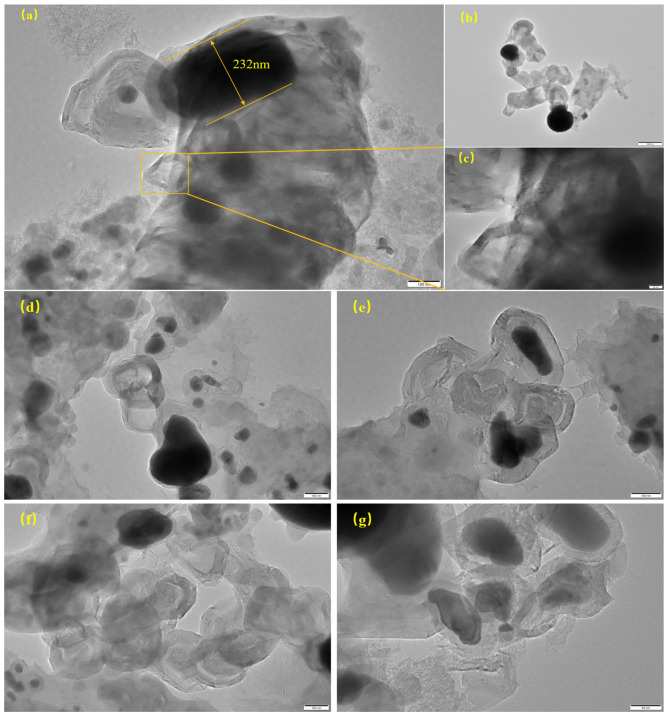
Transmission electron microscopy analysis of the catalyst after reaction at 850 °C: (**a**) Low-magnification TEM of a large Fe particle (232 nm) in the carbon matrix; (**b**) Transmission electron microscopy of spherical iron particles and bamboo shaped carbon nanotubes; (**c**) Magnified view of the marked region in (**a**); (**d**–**g**) TEM showed carbon nanotubes in the biochar carrier.

**Figure 14:**
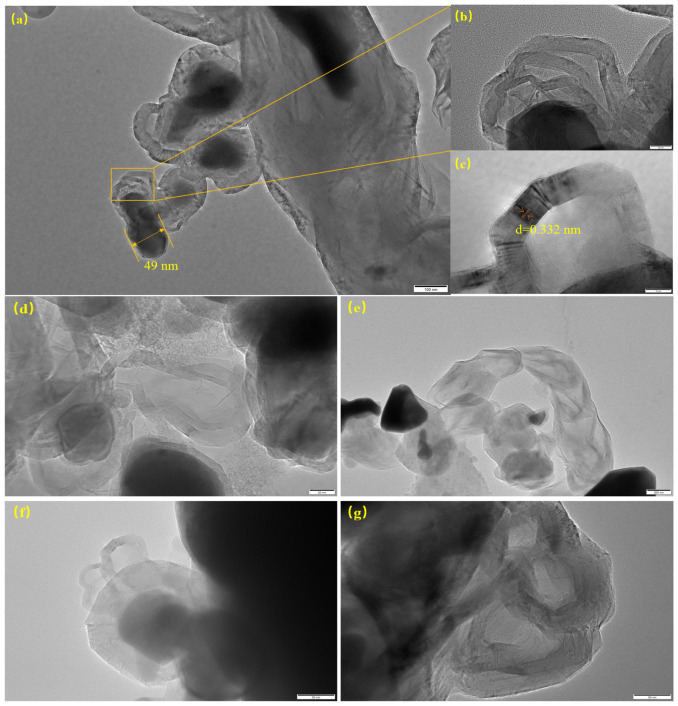
TEM characterization of the catalyst after reaction at 900 °C: (**a**) TEM image of a 49 nm Fe particle; (**b**) An enlarged view of figure (**a**) showing carbon nanotubes around iron particles; (**c**) Lamellar spacing of graphite carbon under HRTEM; (**d**–**g**) TEM showed carbon nanotubes in the biochar carrier.

TEM characterization was performed on the catalysts after reaction at 850 °C and 900 °C, with the results displayed in Figure 13 and Figure 14. As shown in the images, carbon nanostructures with varying dimensions and morphologies are observed on the post-reaction catalysts. Studies have shown that both metallic iron and iron carbide can catalyze the growth of carbon nanotubes (CNTs) with different structures and diameters [34]. Previous studies have shown that iron first decomposes methane into amorphous carbon, followed by the formation of Fe_3_C (iron carbide). When Fe_3_C becomes saturated with carbon, the carbon separates out as graphite [35]. This graphite forms CNTs. Finally, the graphite coats the iron particles, deactivating the catalyst. This matches our test results. From Figure 13a, a large, elongated iron particle about 232 nanometers in size can be found. It is surrounded by layered carbon material. This eventually causes the catalyst to become inactive. This particle, surrounded by layered carbon, formed a typical ‘core-shell’ structure. This phenomenon reveals that the primary deactivation mechanism at this temperature is catalyst deactivation caused by encapsulation of ‘onion-like carbon’. Figure 13c shows that iron particles facilitate the growth of carbon nanofilaments. Some nanotubes with special shapes are also found. These include “open-ended” and “chain-like” nanotubes, as seen in Figure 13b. Studies show that temperature, iron particle size, and the catalyst support all matter. These factors significantly influence the morphology of the carbon materials, such as carbon nanotubes, produced from methane cracking [36,37]. Both metallic iron and iron carbide can act as catalysts. They produce carbon nanotubes with different structures and widths [38]. Figure 14b presents a magnified view of CNTs with a diameter of about 18 nm from Figure 14a, surrounded by several tens of graphitic layers (approximately 40 layers here).

Measurement of the metal cluster size indicates a particle diameter of around 49 nm. Figure 14a shows a pear-shaped metal catalyst particle with a size of approximately 49 nm stably attached to the tip of a carbon nanotube with an outer diameter of about 18 nm. The great advantage of this growth mode is that the catalyst particle can remain exposed to the methane atmosphere at all times, thereby endowing the catalyst with a longer catalytic lifetime at 900 °C. The interlayer spacing of the carbon-generated product was measured to be 0.332 nm (Figure 14c), which is close to the interlamellar spacing of graphite. Figure 14e shows how the “open-ended” carbon nanotubes grow via a tip-growth process, with the catalyst particles remaining firmly attached to the nanotube tips. In addition, the flow of methane and nitrogen gases may contribute to the irregular morphology of the metal particles.

### 2.3. The Porous Structure and Catalytic Properties of Biomass Carbon

The porous structure of biomass carbon mainly comes from the cellular structure of biomass raw materials themselves and the release of volatile components during pyrolysis [39]. The porous structure of biomass carbon is one of the key factors affecting the catalytic performance of biomass carbon [39]. Biocarbon catalysts with suitable pore size distribution can provide more accessible active sites, promoting the diffusion and adsorption of reactant molecules.

The pore structure of biomass carbon usually includes three types: micropores, mesopores, and macropores. Micropores mainly provide high specific surface area. Mesopores and macropores facilitate mass transfer and diffusion of reactants.

Biomass carbon not only has a certain catalytic activity for methane, but also its rich porous structure facilitates the highly dispersed metal active components. The functional groups on the surface of biochar can form strong interactions with metals, inhibiting the migration and aggregation of metal nanoparticles and achieving synergistic catalytic effects.

### 2.4. Impact of Biomass Carbon as a Metal Carrier on Catalytic Cracking of Methane

**Figure 15:**
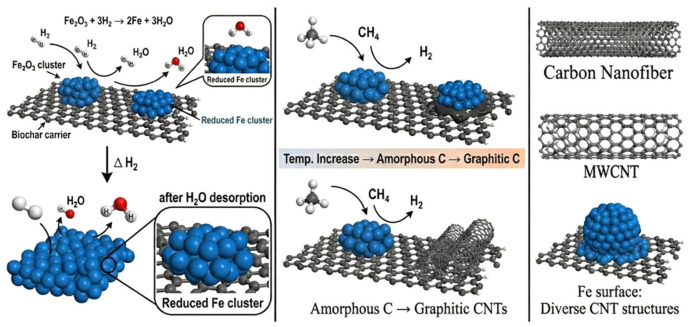
Mechanism diagram of catalytic cracking of methane by biomass carbon supported metal catalyst (in the figure, the blue sphere is Fe atom, the black sphere is C atom, and the red sphere is O atom).

In methane catalytic cracking reactions, biomass carbon, as a green and efficient functional carrier, exhibits unique properties superior to traditional inorganic oxides such as Al_2_O_3_ and SiO_2_. In terms of chemical composition, biomass carbon not only enhances the adsorption capacity of metal precursors, but also regulates the electronic structure of active sites through strong metal carrier interactions, thereby reducing the activation energy of C-H bonds. From the perspective of physical structure, biomass carbon has a developed hierarchical pore structure and a huge specific surface area, which provides good physical support for the uniform dispersion of transition metal active components (such as Ni, Fe), effectively suppressing the thermal sintering phenomenon of active sites at high temperatures. The mechanism diagram of methane cracking catalyzed by biomass carbon-loaded metal catalyst is shown in Figure 15. Firstly, methane molecules diffuse to the surface of metal active sites and undergo a gradual dehydrogenation process on the metal surface. In this process, the breaking of the first C-H bond is usually considered as the rate controlling step of the entire reaction. The released hydrogen atoms bind to the metal surface and desorb in the form of high-purity H_2_.

Secondly, the active carbon atoms generated by dissociation determine the stability of the catalyst. In the presence of biomass carbon carriers, activated carbon atoms follow a “dissolution diffusion precipitation” mechanism. The generated carbon atoms first dissolve and infiltrate into the interior of the metal nanoparticles, or diffuse along the metal surface. Due to the carbon concentration gradient and temperature gradient between the front and back ends of metal particles, carbon atoms will migrate towards the lower concentration end. As a carrier, biomass carbon’s developed hierarchical pore structure not only provides a high specific surface area to achieve high dispersion of metal particles, but also facilitates the migration of carbon atoms.

Finally, when the carbon inside the metal particles reaches saturation, carbon atoms nucleate at the metal–support interface or on the back of the metal, and deposit as either carbon nanotubes (CNTs) or carbon nanofibers (CNFs).

### 2.5. Application Prospects

Among various hydrogen production technologies, methane catalytic cracking can simultaneously produce high-purity hydrogen gas and solid carbon materials with high added value, with direct carbon dioxide emissions during the process [40]. However, traditional metal catalysts such as nickel, iron, cobalt, etc., are prone to rapid deactivation due to carbon deposition during the reaction process. The high cost of conventional commercial carriers such as Al_2_O_3_ and SiO_2_ limits the industrial application of this technology. Therefore, the development of low-cost, efficient, and excellent anti carbon deposition catalyst systems is of great research significance.

Biomass carbon comes from agricultural and forestry waste such as straw and sawdust, with a wide range of sources and low cost [41]. Converting biomass into catalyst carriers not only solves the environmental pressure of waste disposal, but also significantly reduces the preparation cost of hydrogen production catalysts. Secondly, biomass carbon has a rich pore structure and a huge specific surface area, which can effectively disperse metal active components and suppress high-temperature sintering of metal particles. The abundant oxygen-containing functional groups and alkali metal ash on the surface of biomass carbon can produce a synergistic effect with the loaded metals. More importantly, biomass carbon itself is a carbon material that has good compatibility with the sedimentary carbon produced by cracking. This enables the carbon nanotubes or carbon fibers generated by the reaction to grow on biomass carbon as the skeleton, delaying the deactivation time of the catalyst.

## 3. Experiments and Analytical Testing

### 3.1. Experimental Materials

To prepare the catalyst for investigating its performance in methane cracking, corn stalks with a particle size of 150–250 μm as the biomass feedstock. All chemical reagents used in the experiments, including absolute ethanol, KOH, and Fe(NO_3_)_3_·9H_2_O, were analytical grade without further purification. The detailed information of reagents and manufacturers is listed in Table 3.

### 3.2. Preparation of Supported Catalysts

Corn stalk powder was first transformed into biochar via high-temperature pyrolysis. The biochar was treated with KOH activation to generate a hierarchical porous structure, which facilitates subsequent metal immobilization on the biochar support. The detailed experimental steps are as follows.

First, 15 g of corn stalks powder with particles sized 150–250 μm were spread evenly in a quartz boat. Then, the boat was placed into a tube pyrolysis furnace to carry out carbonization. The carbonization process was conducted at 1000 °C for 30 min under a continuous nitrogen atmosphere (2 L/min) to ensure an inert environment. The pyrolysis furnace was heated at a ramp rate of 10 °C/min. Upon completion of pyrolysis, the quartz boat was displaced to the cooling zone of the furnace while maintaining nitrogen flow, allowing the sample to cool naturally to below 50 °C. The resulting material was collected and designated as pristine corn-stalk-derived biochar.

**Figure 16:**
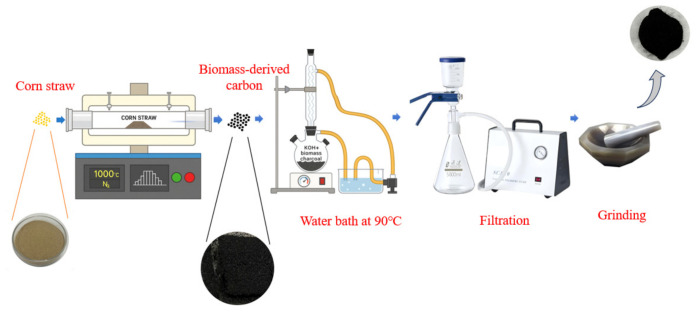
Method for the Pyrolysis and Activation Preparation of Biochar.

For chemical activation, an aqueous potassium hydroxide solution was prepared by dissolving 10 g of KOH pellets in 90 mL of deionized water with vigorous stirring. Subsequently, 5 g of the as-prepared biochar was immersed in the KOH solution and subjected to activation at 90 °C. The activation treatment was maintained for 2.5 h, during which a reflux condenser was employed to prevent solvent evaporation and ensure a stable alkali concentration throughout the process. After the reaction was complete, the liquid was filtered, and the solid product was collected. During filtration, deionized water was used to wash the sample until it reached neutrality. The collected sample was then dried for 12 h. The preparation process of the biochar is showed in Figure 16.

**Figure 17:**
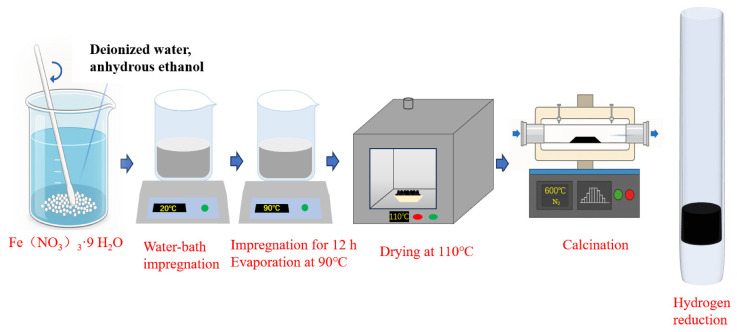
Catalyst preparation process.

First, 5.42 g of Fe(NO_3_)_3_·9H_2_O sample was dissolved in 40 mL of deionized water and 10 mL of anhydrous ethanol solution. The biochar (3 g) was impregnated in the solution for 12 h, then the solvent was evaporated in a water bath (temperature 70 °C), and finally, the sample was dried in a 110 °C drying oven for 12 h. The as-prepared biochar-supported catalyst was initially subjected to calcination at 600 °C for a duration of 2 h in a nitrogen environment and subsequently reduced under a hydrogen atmosphere at 600 °C for 2.5 h to yield the final catalytic material. The metallic Fe loading in the final catalyst was controlled at 20 wt%. The preparation procedure is illustrated in Figure 17.

### 3.3. Methane Decomposition Experiment

**Figure 18:**
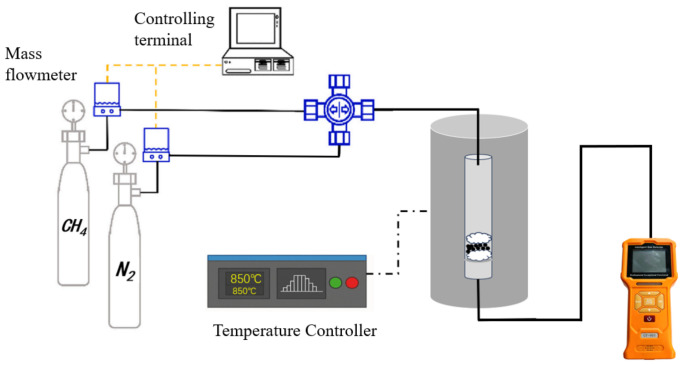
Schematic of the Methane Cracking Setup.

The diagram of the methane cracking setup is shown in Figure 18, which includes gas cylinders, a flow control system, a reactor, and a system to monitor the gas flow. Subsequently, 0.5 g of biochar-supported Fe catalyst was added to the reactor. Before the reaction begins, the air inside the reactor is displaced by introducing a mixture of methane and N_2_ gas (methane volume concentration of 10%) at a flow rate of 50 mL/min. The reactor was then heated to 800 °C, 850 °C, 900 °C, and 950 °C respectively. Methane concentration was monitored in real time using an online methane analyzer (Shenzhen Korno Electronic Technology Co., Ltd., Shenzhen, China). The outlet methane concentration was determined online using a GT-903 multi-gas detector (Shenzhen Korno Electronic Technology Co., Ltd., Shenzhen, China), which is based on the NDIR detection principle. The methane measurement range is 0–50 vol.%, with a resolution of 0.01 vol.%. The instrument was calibrated with standard gases before use, and real-time CH_4_ concentrations were recorded throughout the reaction.

### 3.4. Analytical Characterization

In this work, methane conversion (both real-time and total conversion) was selected as the key index to evaluate the catalytic performance of methane decomposition. Due to the limitation of the gas detection equipment, hydrogen concentration could not be measured online. Based on the stoichiometry of methane decomposition (CH_4_ → C + 2H_2_), the methane conversion is directly proportional to the hydrogen yield under the tested conditions, so it can effectively reflect the reaction performance.

(1) Instantaneous methane conversion volume (mL):

$$X\left(\mathrm{mL}\right)\;=\;\frac{A_{{\mathrm{CH}}_{4,}\mathrm{in}}\;-\;50\;\times \;C-C\;\times \;A_{{\mathrm{CH}}_{4,}\mathrm{in}}}{12\;\times \;C\;+\;6}$$

(2) Instantaneous methane conversion rate (%):

$$P_{{\mathrm{CH}}_{4}}\left(\%\right)\;=\frac{A_{{\mathrm{CH}}_{4,}\mathrm{in}}-50\;\times \;C-C\;\times \;A_{{\mathrm{CH}}_{4,}\mathrm{in}}}{12\;\times \;C\;\times \;A_{{\mathrm{CH}}_{4,}\mathrm{in}}\;+\;6\;\times \;A_{{\mathrm{CH}}_{4,}\mathrm{in}}}\times 100\%$$

(3) Total methane conversion rate (%):

$$X_{{\mathrm{CH}}_{4}}\left(\%\right)=\frac{V_{{\mathrm{CH}}_{4},\mathrm{total}}}{F_{{\mathrm{CH}}_{4},\mathrm{in}}}\;\times \;100\%$$

where $A_{{\mathrm{CH}}_{4},\mathrm{in}}$ and $A_{{\mathrm{CH}}_{4},\mathrm{out}}$ represent the volumetric flow rates (mL/min) of methane at the reactor inlet and outlet, respectively.

(4) Specific Surface Area and Porosity Analysis (BET): The specific surface area, pore volume, and pore size distribution of the catalyst were determined using a Micromeritics ASAP 2460 analyzer (Micromeritics Instrument Corporation, Norcross, GA, USA) [42].

(5) Scanning Electron Microscopy (SEM): The fresh and spent catalysts were examined via EVO18 scanning electron microscopy to evaluate how reaction temperature influences the products generated from methane catalytic cracking.

(6) X-Ray Diffraction (XRD): To study the evolution of catalyst composition throughout the reaction process, a Rigaku Smartlab X-ray diffractometer was used to analyze the catalyst. The radiation source was Cu Kα rays (γ = 0.15418 nm).

(7) Raman Spectroscopy: It was performed on the solid carbon products after the reaction to study the degree of graphitization, in order to assess the structural order and defect density of the samples.

(8) High-Resolution Transmission Electron Microscopy (HRTEM): The catalytic reaction was characterized using a 2100F/Talos high-resolution transmission electron microscope (Thermo Fisher Scientific, Waltham, MA, USA). It can observe the morphology of the carbon formed after the reaction and the changes in the appearance of the catalyst itself.

## 4. Conclusions

(1) The higher the temperature, the better the methane conversion effect, with higher reaction efficiency and cumulative conversion levels. At 950 °C, the catalyst exhibited optimal performance, achieving a peak conversion rate of 85% while maintaining a stable conversion of approximately 55% after prolonged reaction; the overall conversion reached 57.6%.

(2) After the catalyst was used in the methane reaction, its surface area became much smaller. The tiny pores disappeared. The medium-sized pores also decreased slightly. At the same time, the average pore size grew significantly.

(3) With increasing reaction temperature, *I_D_*_1_*/I_G_* value showed a distinct monotonic decreasing trend. Within the studied temperature range, higher temperatures significantly promoted the transformation of the solid carbon products from highly obstructive amorphous carbon toward more ordered graphitic carbon.

(4) The main solid product obtained from methane catalytic cracking is carbon deposits formed in situ on the surface of the biochar-supported Fe catalyst. Based on XRD, SEM, and TEM characterization results, the deposited carbon is mainly composed of disordered carbon layers and filamentous carbon, accompanied by a small amount of carbon nanotubes.

(5) However, it should be noted that the catalyst deactivation caused by carbon deposition is still a key problem, and the recyclability and regeneration performance of the catalyst need to be further studied in future work to promote its practical application.

## Figures and Tables

**Table 1:** Pore structure characteristics of the biochar samples.

Biochar Samples	BET Surface Area (m^2^/g)	Amicro (m^2^/g)	Ameso (m^2^/g)	Vtotal (cm^3^/g)	Vmicro (cm^3^/g)	Vmeso (cm^3^/g)	Average Pore Size (nm)	Average Mesopore Size (nm)
600 °C	327	211.91	114.727	0.2426	0.08549	0.15711	2.971	5.478
800 °C	64	0.000	63.736	0.1244	0.000	0.1244	7.809	7.807
850 °C	71	0.000	70.790	0.1254	0.000	0.1254	7.086	7.086
900 °C	45	0.000	45.422	0.1059	0.000	0.1059	9.326	9.326
950 °C	64	0.000	63.956	0.1374	0.000	0.1374	8.590	8.593

**Table 2:** Parameters of peak deconvolution and fitting for the Raman spectrum (1000–2000 cm^−1^ region) of the catalyst sample.

Peak Name	Peak Position (cm^−1^)	Description of Raman Peak Deconvolution	Peak Type
G	1591	Graphite; quarter-ring breathing of aromatic systems; alkene C=C	sp^2^
D3	1523	Aromatic clusters containing 3–5 rings; amorphous carbon structures	sp^2^
V1	1480	Methylene or methyl groups; semi-ring breathing of aromatic systems; amorphous carbon structures	sp^2^, sp^3^
D1	1346	C–C and C=C bonds in polyolefin-like structures	sp^2^
D4	1232	Aryl-alkyl ethers; aromatic (aliphatic) ethers	sp^2^, sp^3^
S1	1160	Hexagonal diamond-like sp^3^ carbon; aromatic ring C-H	sp^2^, sp^3^

**Table 3:** Experimental Raw Materials and Main Reagents.

Reagent Name	Specifications	Manufacturer
KOH	Analytically pure	Tianjin Tianli Chemical Reagent Co., Ltd., Tianjin, China
Fe(NO_3_)_3_·9H_2_O	Analytically pure	Tianjin Tianli Chemical Reagent Co., Ltd., Tianjin, China
C_2_H_5_OH	Analytically pure	Tianjin New Technology Industrial Park Kemao Chemical Reagent Co., Ltd., Tianjin, China
CH_4_	99.99 vol.%	Harbin Jiechun Gas Co., Ltd., Harbin, Heilongjiang, China
N_2_	99.99 vol.%	Harbin Jiechun Gas Co., Ltd., Harbin, Heilongjiang, China
H_2_	99.99 vol.%	Hydrogen generator, Shandong Saikesaisi Hydrogen Energy Co., Ltd., Jinan, China
deionized water	Analytically pure	Self-made in laboratory
quartz cotton	Analytically pure	Shanghai Xinhu Experimental Equipment Co., Ltd., Shanghai, China

## Data Availability

The raw data supporting the results of this study are available from the corresponding author upon reasonable request.
